# Mr. Philips, on Vaccina, &c.

**Published:** 1801-06

**Authors:** C. T. Philips

**Affiliations:** Painswick


### *To the Editors of the Medical and Physical Journal.*

Gentlemen,

If you deem the following cases deserving a place in your valuable Journal, they are at your disposal, and their insertion will greatly oblige, Your's, &c.

*Painswick, May* 3, 1801. C. T. PHILIPS.

---

Though the mass of evidence brought forward by many of the most eminent professional characters, not only of this but of other countries, respecting the superior advantages the Vaccine Disease possesses over the Variolous, and which, to unprejudiced minds, leaves not a doubt; yet still, as there are many who are decidedly decidedly inimical to, and prejudiced againſt it, every ray of information which will tend to ſerve his fellow-creatures and the cauſe of truth, every man is bound by a reciprocal duty to bring forward; and as your excellent work particularly promotes theſe intentions, permit me to offer my ſmall tribute of evidence in favour of the inoculated cow-pox, and to mention an irregularity which lately fell under my obſervation.

February 23d, I inoculated the Rev. Mr. F.'s two younger children and ſervant girl with the vaccine virus. 25th, Inflammation nearly obliterated. 26th, I was induced to repeat the operation on the ſervant girl, as there did not remain the leaſt veſtige of inflammation either in her or the childrens' arms.

From the 26th to March 2d, I examined her arm daily, (Sunday excepted), but the inflammation was ſo trivial as to lead me (as well as Mr. F.) to conclude the infection had not taken, and I in conſequence wrote to the perſon who favoured me with the virus, to inform him of the diſappointment I felt, and to requeſt a freſh ſupply, conceiving its failure might depend upon its ſtaleneſs. To my great ſurpriſe, on Saturday following, the 7th, I was deſired to viſit her, and upon inſpecting the arm, found the infection had taken, and to appearance the puſtule was characteriſtic of the 8th day of the diſeaſe, though ten from the *ſecond* inoculation. From her I immediately inſerted matter into each of the childrens' arms, who both went through the diſeaſe with little or no indiſpoſition, though the progreſſive appearances upon the arms were ſufficiently diſtinctive to relieve me from any anxiety reſpecting its being the genuine diſeaſe.

April 22d, I inoculated the above three perſons with variolous matter, taken the day before from a healthy young woman labouring under the ſmall-pox from inoculation; and to give the experiment every poſſible chance, I inſerted a conſiderable quantity of matter. 23d, Inflammation in all, ſufficient to denote infection, had they been capable of taking the diſeaſe. 24th, Inciſions ſtill more inflamed. 25th, Inflammation much leſs in youngeſt child and ſervant, rather more in the eldeſt's arms. 26th, Did not ſee them. 27th, Inflammation declining in the eldeſt, and nearly gone in the youngeſt child and ſervant. 28th, In each the inflammation rapidly dying away. Saturday, May 2d, The arm of each perfectly well.

In all, I have inoculated for the cow-pox 33; and from the experience I have thus far had of the diſeaſe, I feel fully ſatisfied of its moſt decided ſuperiority over the ſmall-pox. My patients, in general, were but little indiſpoſed, and I have not experienced one alarming, troubleſome, or puſtular caſe.

To

To the above cafes I have to offer one of a fpafmodic contraction of the fphincter veficæ urinariæ and urethra fuccefsfully treated with the tinctura ferri muriati.

Enoch Daniels, a child fix years old, was brought to me, labouring under moft excruciating pain upon voiding his urine, which would fuddenly ftop when he had difcharged about one half. The pain ufually feized him about a minute before, and continued about the fame time after making his water, during which time his fufferings were moft urgent; fo great, that he would fcream to be heard to a confiderable diftance, grafping forcibly the penis, and incurvating his body. He was perfectly eafy in the intervals, and in all other refpects tolerably well; though fome little time before he had a fever, of which he was cured with the faline antimonial mixture. Not being perfectly well fatisfied at firft, as to the nature of his cafe, and fufpecting he might be troubled with a calculous affection, I ordered him the Aq. kali pur. gt. v. bis in die ex poculo decoct. rad. althææ, from which he derived fome little benefit. Still further to fatisfy myfelf as to the prefence of calculi, I founded him, but could not find any thing to juftify an opinion upon that head and his fufferings ftill continuing, I was induced to try the above-mentioned remedy, recollecting a cafe fpoken of by Mr. Cline in his lectures, and to which I conceived the prefent very analogous as to fymptoms. Mr. C. I think, in his cafe, recommended ten drops of the tincture to be taken every ten minutes during the fpafm. In this cafe, I defired three drops to be taken in a little water every ten minutes, upon the coming on of the fpafm, and continued till it fubfided. Upon feeing him the third day from beginning the tincture, I found him much better; continuing the fame dofe a few days longer with decided good effect, I ordered the tincture to be increafed two drops, from which he experienced ftill greater relief. After taking about three drachms of the tincture, he was fo much better as to have little or no pain upon making water, and that only occafionally. I now ordered gt. v. bis terve in die; from a continuance of which I have the pleafure to fay, he is fo far recovered as to bid fair for a perfect cure.

He was fo far recovered at one time as to lofe all pain; but relinquifhing his medicine too foon, he had a trifling relapfe; however, by reforting to it again, he experienced its former efficacy.

---

Remarks

